# A Case of Mauriac Syndrome: A Teenage Girl With Poorly Controlled Diabetes

**DOI:** 10.7759/cureus.64748

**Published:** 2024-07-17

**Authors:** Austin Kleint, Monica Dussan, Arjun Chandran, Mohammed Salameh

**Affiliations:** 1 Pediatrics, Baylor College of Medicine, San Antonio, USA; 2 Pediatric Endocrinology, Baylor College of Medicine, San Antonio, USA; 3 Pediatric Critical Care Medicine, Baylor College of Medicine, San Antonio, USA

**Keywords:** type 1 diabetes, hepatomegaly, poorly controlled diabetes, short stature (ss), glycogenic hepatopathy, mauriac syndrome

## Abstract

Mauriac syndrome is a rare complication of longstanding, poorly controlled type 1 diabetes in pediatric patients. Mauriac syndrome is characterized by hepatomegaly and growth retardation.

This case report discusses a 14-year-old girl with persistent, poorly controlled type 1 diabetes mellitus (T1DM) admitted to the pediatric intensive care unit (PICU), where she was ultimately diagnosed with Mauriac syndrome. The patient presented with severe hypoglycemia and a history of multiple admissions for diabetes ketoacidosis (DKA) despite insulin therapy. The patient had a history of poor glycemic control and growth retardation, and on physical exam, she was found to have hepatomegaly. Based on clinical presentation, history of poorly controlled diabetes hepatomegaly, imaging, and laboratory findings, the diagnosis of Mauriac syndrome was made. Management of the patient included diabetes education, optimizing insulin therapy, nutritional support, and closely monitoring labs in a multi-disciplinary approach. The patient responded well to insulin therapy and was started on closed-loop insulin administration. Liver enzymes and hepatomegaly normalized, and growth parameters improved over the subsequent months.

This case emphasizes the importance of early recognition, monitoring, and management of an extremely rare syndrome that is crucial in preventing the short-term complications of lactic acidosis and rapidly progressing retinopathy and the long-term complications of hepatic dysfunction and growth impairment.

## Introduction

Mauriac syndrome is a rare complication of type 1 diabetes mellitus (T1DM) in pediatric patients, which manifests with hepatomegaly and growth retardation due to chronic poor glycemic control [1-8]. The glycogen deposits built in the hepatocytes of these patients result during episodes of persistent hyperglycemia, where glucose gets converted to glycogen independently of insulin, which mediates the conversion of glucose to glycogen in the liver [1,8,9]. The causes of delayed growth and delayed puberty are still not well understood [9]. Dysregulation of growth hormone, insulin growth factor, and hypercortisolism are some of the proposed mechanisms [9]. Patients diagnosed with Mauriac syndrome are at high risk of developing lactic acidosis and rapidly progressing retinopathy with acute management of hyperglycemia [10-13].

The objective of this case report is to highlight the rarity of Mauriac syndrome, outline clinical features, diagnostic considerations, and therapeutic interventions, discuss pitfalls of management, and raise awareness of potentially poor outcomes closely related to Mauriac syndrome. This case report aims to contribute valuable insights into the management of Mauriac syndrome in pediatric diabetes care and the importance of early recognition and optimized management in mitigating complications.

## Case presentation

**Figure 1:**
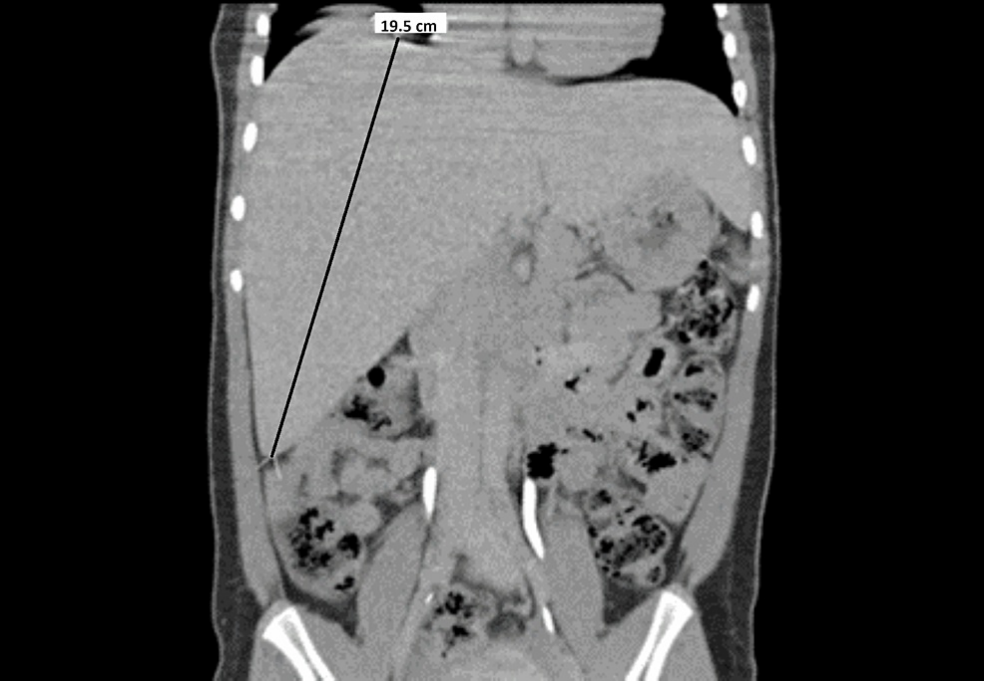
Hepatomegaly with the liver measuring 19.5 cm

A 14-year-old female with a history of poorly controlled type 1 diabetes mellitus (T1DM), short stature (Z score -2.16), hepatomegaly, and elevated liver enzymes, was admitted to the PICU with altered mental status in the setting of severe hypoglycemia. The patient was diagnosed with T1DM at four years of age. Before hospitalization, the patient had episodes of hypo and hyperglycemia in the setting of insulin non-compliance with erratic boluses of long and short-acting insulin. The patient was hospitalized 14 times for diabetes ketoacidosis (DKA) over 4.5 years before the current hospitalization. Medication non-compliance made adjusting her insulin regimen difficult in the outpatient setting. Her hospital course was notable for fluctuating glucose levels, with hyperglycemic events during the day and hypoglycemic events overnight. Abdominal imaging showed an enlarged liver, with a “diffuse coarse/grainy and mildly echogenic appearance of the hepatic parenchyma” (Figure 1).

An extremely elevated insulin and low C-peptide level obtained upon arrival at the hospital was consistent with an inappropriate insulin dose administered at home. The patient’s hemoglobin A1C was 14.2%. The lactate level was elevated upon admission. Invitae Mucopolysaccharidoses Plus (MPS+) and Glycogen Storage Disorder genetic testing were negative, including a normal PHKG2 gene. Relevant and important laboratory tests on admission can be seen in Table 1.

**Table 1:** Relevant laboratory tests upon admission

Laboratory Test	Results	Normal Range
Potassium	4.5 mmol/L	3.5-5.1 mmol/L
Bicarbonate	21 mmol/L	20-28 mmol/L
Glucose	345 mg/dL	60-100 mg/dL
Lactic Acid	3.2 mmol/L	0.5-2.2 mmol/L
Aspartate Amino Transferase	145 U/L	5-34 U/L
Alanine Aminotransferase	152 U/L	0-55 U/L
Alkaline Phosphatase	146 U/L	20-170 U/L
Hemoglobin A1C	14.2 %	4.2-5.8 %
Insulin Level	>300.0 uU/mL	<35 uU/mL
C-Peptide	0.1 ng/mL	0.5-3.3 ng/mL
Insulin-Like Growth Factor-Binding Protein-3	8690 ng/mL	2654-6680 ng/mL

**Figure 2:**
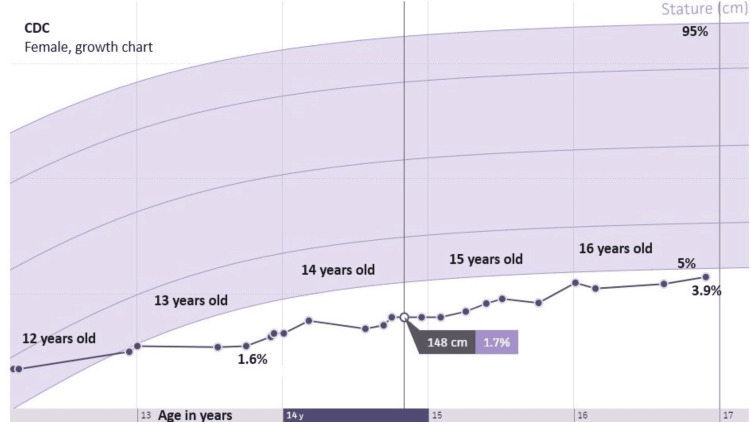
CDC growth chart of the hospitalized patient from the outpatient endocrinology clinic CDC: Centers for Disease Control and Prevention Source: https://www.cdc.gov/growthcharts/cdc-growth-charts.htm

The patient was found to be heterozygous for Alpha-1 antitrypsin (A1AT) deficiency with a rare allele of unknown significance. The patient’s glucose was adequately controlled with a new insulin regimen on day 4 of hospitalization, and she was discharged home. The patient was previously diagnosed with bilateral cataracts but has not developed retinopathy. Insulin-like growth factor-binding protein 3 was found to be mildly elevated for a Tanner stage, with a chronological age of 14 years, 8 months, and a bone age of 14 years, 0 months. After hospital discharge, the patient was transitioned to a hybrid closed-loop system. The patient’s A1C decreased to 8.7% four months after starting the closed loop, marking the first time with an A1C below 14% in over 3 years while followed within our institution. Upon improvement of glycemic control, the patient’s abdominal distention and hepatomegaly significantly improved by physical examination, with liver palpation one centimeter below the right costal margin where it had previously been five centimeters. The patient's liver enzymes have also returned within normal limits following improvement of glycemic control. The patient's short stature improved following glycemic control going from 1.7% to 3.9% on the Centers for Disease Control and Prevention (CDC) female growth chart (Figure 2).

## Discussion

Glycogen hepatopathy and stunted growth are well-recognized consequences of poorly controlled T1DM [1-8]. This pathology was a frequent occurrence in the early 1900s and was first described by Pierre Mauriac in 1930 [14]. It is characterized by hepatomegaly, cushingoid appearance, and growth failure in poorly controlled T1DM [14]. Experimentation of insulin on human subjects began in 1922 and shortly after that, the production and distribution of the lifesaving medication began [15]. Improvements in insulin have continued throughout the twenty-first century [15]. Monitoring blood glucose levels remained difficult until self-monitored blood glucose became the standard of care in the 1980s [16]. With the improvements in self-monitored blood glucose and insulin, Mauriac syndrome has become increasingly rare [17]. The pathophysiology of hepatic glycogenosis is not fully understood but is likely multifactorial from episodes of hyperinsulinemia and hyperglycemia [1,8,9]. The deposition of glycogen stores in the liver during hyperinsulin episodes is likely secondary to biochemical pathways leading to glycogenesis and inhibition of glycogenolysis via the activation of glycogen-synthase and glucokinase with the inhibition of glucose-6-phosphatase [8,10,18]. Elevated lactate levels in Mauriac syndrome can be explained with glycolysis when the Krebs cycle is saturated, and pyruvate undergoes lactic acid fermentation (Type-B hyperlactatemia) [10-12]. Closer monitoring of lactate levels is indicated with the management of DKA in these patients [10-12]. The cause of short stature, delayed puberty, and cushingoid body habitus is hypothesized to be from dysregulation of cortisol, growth hormone, and insulin-like growth factor from fluctuations of hyperglycemia and hypoglycemia [8].

The predisposition of Mauriac syndrome is unknown to be genetic or acquired given not all patients with poorly controlled T1DM develop Mauriac syndrome [9]. In 2016, a heterozygous genetic mutation in PHKG2 providing a subunit of glycogen phosphorylase kinase enzyme complex was discovered in a patient who was diagnosed with Mauriac syndrome [9]. The PHKG2 mutation is the only mutation discovered when cross-referenced with the online Mendelian inheritance in a man with a genetic predisposition for Mauriac syndrome [9]. Genetic testing appears to be promising to further characterize this complex syndrome. The patient’s A1AT level was mildly low in the setting of normal inflammatory markers, and the heterozygous Alpha-1 antitrypsin (A1AT) mutation is likely non-contributary to our patient given the lower end of normal A1AT level and improvement of hepatomegaly following improvement of glycemic control [19].

A liver biopsy is not necessary for diagnostic evaluation but may play a role if the diagnosis is unclear or if there is a concern for other pathology [1,8,17,20]. This patient did not develop retinopathy, but caution should be taken with rapidly controlling glucose in patients with Mauriac syndrome given the increased risk of rapid progression of retinal disease [13]. Mauriac syndrome, characterized by the combination of glycogenic hepatopathy and stunted growth in the setting of poorly controlled T1DM, has become increasingly rare with the availability of multiple daily insulin injections, insulin pumps, and closed-loop systems in developed countries but remains underdiagnosed in patients with long-standing poorly controlled T1DM [1,2,8,17,20].

Given the rarity of Mauriac syndrome, the goal of this case is to enhance clinician awareness of the syndrome, the associated complications, and the reversibility with appropriate management. Early and appropriate management is imperative to aid in appropriate monitoring and interventions of short- and long-term complications. Moving forward, continued awareness and reporting of cases are essential for appropriate treatment and advancements related to Mauriac syndrome. Future research efforts are warranted to understand the underlying pathophysiology and the possible genetic predispositions.

## Conclusions

Mauriac syndrome presents with growth failure, delayed puberty, and hepatomegaly in pediatric patients with insufficient diabetic control. Patients are at higher risk for both lactic acidosis and rapidly progressing retinopathy with an improvement in glycemic control. Given the short- and long-term complications that can result from this disease and the potential reversibility with proper glycemic management, early recognition of the syndrome, appropriate multidisciplinary management, and close follow-up will aid in improving outcomes.
